# Machine Learning Models for the Prediction of Postpartum Depression: Application and Comparison Based on a Cohort Study

**DOI:** 10.2196/15516

**Published:** 2020-04-30

**Authors:** Weina Zhang, Han Liu, Vincent Michael Bernard Silenzio, Peiyuan Qiu, Wenjie Gong

**Affiliations:** 1 XiangYa School of Public Health Central South University Changsha China; 2 Sanofi Global Research and Design Operations Center Chengdu China; 3 Urban-Global Public Health Rutgers School of Public Health Rutgers, The State University of New Jersey Newark, NJ United States; 4 West China School of Public Health Sichuan University Chengdu China

**Keywords:** depression, postpartum, machine learning, support vector machine, random forest, prediction model

## Abstract

**Background:**

Postpartum depression (PPD) is a serious public health problem. Building a predictive model for PPD using data during pregnancy can facilitate earlier identification and intervention.

**Objective:**

The aims of this study are to compare the effects of four different machine learning models using data during pregnancy to predict PPD and explore which factors in the model are the most important for PPD prediction.

**Methods:**

Information on the pregnancy period from a cohort of 508 women, including demographics, social environmental factors, and mental health, was used as predictors in the models. The Edinburgh Postnatal Depression Scale score within 42 days after delivery was used as the outcome indicator. Using two feature selection methods (expert consultation and random forest-based filter feature selection [FFS-RF]) and two algorithms (support vector machine [SVM] and random forest [RF]), we developed four different machine learning PPD prediction models and compared their prediction effects.

**Results:**

There was no significant difference in the effectiveness of the two feature selection methods in terms of model prediction performance, but 10 fewer factors were selected with the FFS-RF than with the expert consultation method. The model based on SVM and FFS-RF had the best prediction effects (sensitivity=0.69, area under the curve=0.78). In the feature importance ranking output by the RF algorithm, psychological elasticity, depression during the third trimester, and income level were the most important predictors.

**Conclusions:**

In contrast to the expert consultation method, FFS-RF was important in dimension reduction. When the sample size is small, the SVM algorithm is suitable for predicting PPD. In the prevention of PPD, more attention should be paid to the psychological resilience of mothers.

## Introduction

Postpartum depression (PPD) is a serious public health problem that affects 10% to 20% of pregnant women [1-3]. PPD not only adversely affects the physical and mental health of mothers, it is detrimental to the growth and development of infants. In extreme cases even suicide and infanticide may occur [4]. Establishing an effective PPD prediction model that can be used in pregnancy may enable earlier identification, thus, helping health care providers offer more effective management to at-risk patients [5]. Previous studies have explored this possibility and demonstrated its feasibility [6,7].

Machine learning (ML) may be useful in making accurate predictions based on data from multiple sources and has been applied in prediction studies in recent years [8]. There are many predictive factors for PPD including demographics, psychology, and environment [5,9,10]. Assessing risk factors during pregnancy can allow enough time for subsequent interventions. The expert consultation method has often been used to generate guidelines for PPD detection, based on expert opinion and clinical experience. In contrast, ML approaches rely on the use of empirical data to generate prediction models. The key to building good ML models is in the rigorous selection of appropriate features and algorithms. There are two approaches to address the important challenge of feature selection in ML: filter and wrapper [11]. A random forest-based filter feature selection (FFS-RF) algorithm can use the importance score of a so-called random forest (RF) of variables as the evaluation criterion for feature selection, which will identify the subsets of data features that may be most relevant to accurately predict the targeted outcome variable(s) of interest. Such strategies to identify the most relevant data features have proven to be effective ways to explore the risk factors for some diseases [12]. There are two main algorithms used in depression prediction studies, namely, the support vector machine (SVM) and RF algorithms [8]. Depression prediction studies using these two methods have achieved relatively good results [13-15]. SVM is an example of supervised learning. It focuses on minimizing structural risks within the set of available data [16]. It has great advantages in solving high-dimensional modeling problems and performs well in situations that have relatively less available sample data [17]. In contrast, RF models are built using a decision tree as the basic classifier. RF approaches have high classification accuracy, strong inductive capacity, a simple parameter adjustment process, fast calculation speed, relatively low sensitivity to missing data values, and the ability to output feature importance [12,18].

Comparison between those ML methods concerning PPD has not been studied. This study is based on data drawn from a large, ongoing cohort study of pregnant women in the Hunan province of south central China. In this paper we combined the two feature selection methods and the two ML algorithms described above to assess four PPD prediction models using data during pregnancy to compare the effect of PPD prediction models, pick the optimal predictive model, and provide a reference for the development of ML in PPD.

## Methods

### Sampling

This study was part of a larger cohort study. All the data included here is original and previously unpublished. Researchers in the study collected the following measures at a series of 7 visits conducted in the first trimester through 6 weeks postpartum: depression (using the Edinburgh Postnatal Depression Scale [EPDS]), social environment, and psychological and biological factors associated with depression. The study was approved by the institutional review board of the institute of clinical pharmacology of Central South University (ChiCTR-ROC-16009255).

Participants were recruited from two maternity and child care centers in the cities of Changsha and Yiyang in the Hunan province. The former is a major provincial teaching hospital located in Changsha, a city with approximately 8.15 million residents. Yiyang city is a less economically developed area of Hunan province, with approximately 4.39 million residents. Researchers sought to recruit women in the obstetric clinics of the two hospitals from September 2016, to February 2017. The following inclusion criteria were used for participants: woman, age ≥18 years, and gestation period ≤13 weeks (pregnancy weeks are estimated based on the first day of the last menstrual period). All participants signed informed consent. In total, 1126 women were recruited.

### Measures

The following tools were used to collect data.

1. A purpose-built questionnaire, designed for this study and optimized through a pilot survey, was used to collect information including age, education, monthly income level, occupation, marital satisfaction, first pregnancy, folic acid intake, premenstrual syndrome, history of mental health concerns, family history of mental illness, mother's menopausal symptoms, childhood experiences, and life events.
2. The EPDS was used to self-report maternal symptoms of depression [19]. The EPDS is a 10-item self-rated questionnaire, with each item scored from 0 to 3, with a total score ranging from 0 to 30. The Chinese language EPDS used in this study was translated by Wang Yuqiong [20]. The EPDS is the most common PPD screening tool [21,22]. The critical value was 9.5.
3. The Brief Resilience Scale (BRS) was used to determine the level of psychological resilience. The BRS is a 6-item questionnaire that reflects the respondent’s ability to bounce back or recover from stress. The score is the average score of each item. A higher score indicates a stronger strain and adaptability [23].
4. The Pittsburgh Sleep Quality Index (PSQI) is a comprehensive scale that reflects the sleep quality of subjects. It is composed of 7 dimensions: “Sleep Quality”, “Sleep Latency”, “Sleep Duration”, “Sleep Efficiency”, “Sleep Disorders”, “Use of Sleep Medications”, and “Daytime Dysfunction”. The scores of each dimension are summed to obtain the total PSQI score. Higher scores indicate worse sleep quality. According to the total score, sleep quality can be divided into different grades: 6 to 10 indicates “good sleep quality”, 11 to 15 indicates “average sleep quality”, and 16 to 21 indicates “poor sleep quality” [24]. The scale has good reliability and validity [25].
5. The Social Support Rating Scale (SSRS), which was designed by Shuiyuan Xiao [26], was used to measure social support. The SSRS is a 10-item questionnaire with three dimensions, namely, objective support, subjective support, and use of social support. Higher total scores and higher scores for each dimension indicates a better level of social support for an individual.
6. The Generalized Anxiety Disorder-7 (GAD-7) was developed by Spitzer [27]. The score is obtained by summing the scores of 7 items. Most current studies consider a total score of 10 or higher as indicative of anxiety [27,28].

### Procedure

**Figure 1:**
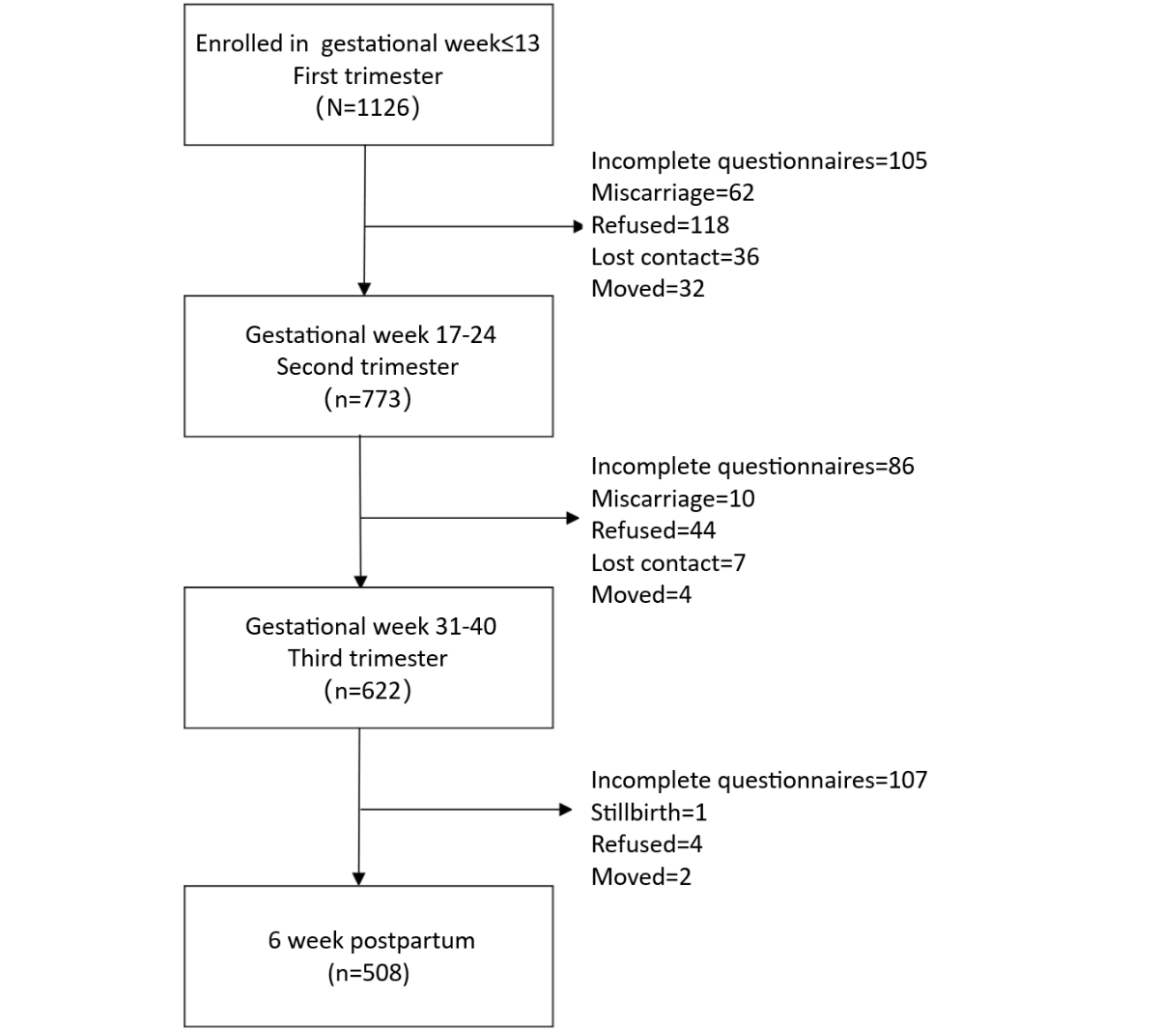
Participant recruitment and response condition.

Seven time points were selected for depression screening, corresponding to the women’s routine obstetric examinations. We divided these into first trimester (gestational week 13 or earlier), second trimester (weeks 17-20 and 21-24), third trimester (weeks 31-32 and 35-40) and postpartum (7 days and 6 weeks postpartum). Except for the first, screening for perinatal depression by EPDS was performed twice for each trimester. If one or more of the EPDS scores was 9.5 or higher for each grouped set of visits, the participant was regarded as at risk for depression during this period. The study questionnaire, BRS, and GAD-7 were assessed during the first trimester, whereas the PSQI was used during the second trimester, and the SSRS during the third trimester. In total, 508 out of 1126 (45.12%) participants completed all screenings (Figure 1).

### Feature Selection

Two simple and easy to implement methods were used for feature selection, namely, the expert consultation and FFS-RF methods. The expert consultation method was used to select clinically relevant factors as appropriate predictors of pre-existing or potential PPD. This was accomplished by consulting experts in the area of obstetrics and gynecology as well as mental health practitioners. The FFS-RF was used to identify proper predictors for PPD. Under this approach, features within a certain bound value range (*P*>.05) were selected as potential predictors and incorporated into the final prediction model.

### Model Development

Of the 508 participants, 75% (381) were randomly selected for model training. Data from the remaining 127 participants was held back for use in model testing and verification. Table 1 shows the model selection scheme. Based on the expert consultation method and FFS-RF method, four PPD prediction models were generated using the SVM and RF algorithms. The parameters of the models were optimized, and the specific parameters are shown in Table 2.

**Table 1:** Names of the postpartum depression prediction models.

Machine learning modeling algorithm	Feature selection method	
	Expert consultation method	FFS-RF^a^
Random forest	E-RF^b^	F-RF^c^
Support vector machine	E-SVM^d^	F-SVM^e^

^a^FFS-RF: filter feature selection based on random forest.

^b^E-RF: model built using the random forest algorithm and expert consultation method.

^c^F-RF: model built using the random forest algorithm and Random forest-based filter feature selection method.

^d^E-SVM: model built using the support vector machine algorithm and expert consultation method.

^e^F-SVM: model built using the support vector machine algorithm and Random forest-based filter feature selection method.

**Table 2:** Optimal parameters for each model.

PPD^a^ prediction model name	Parameter settings
E-RF^b^	n_estimator=300, criterion=entropy, max_features=sqrt
E-SVM^c^	Kernel=linear
F-RF^d^	n_estimator=300, max_features=auto, criterion=gini
F-SVM^e^	Kernel=linear

^a^PPD: postpartum depression.

^b^E-RF: model built using the random forest algorithm and expert consultation method.

^c^E-SVM: model built using the support vector machine algorithm and expert consultation method.

^d^F-RF: model built using the random forest algorithm and Random forest-based filter feature selection method.

^e^F-SVM: model built using the support vector machine algorithm and Random forest-based filter feature section method.

### Evaluation of Model Effects

For the test set, we used the trained models to test and compare their prediction of PPD with real data and created a confusion matrix (Table 3). A series of indicators were obtained of each model. The following index formulas were used.

Accuracy = 
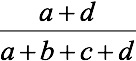


Misclassification rate = 
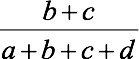


Positive predictive value = 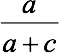


Negative predictive value = 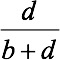


Sensitivity (Sen) = 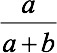


Specificity (Spe) = 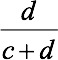


Geometric mean = 
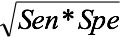


**Table 3:** Confusion matrix.

Predicted Results	Real Results	
	Positive	Negative
Positive	a	c
Negative	b	d

The sensitivity and the receiver operator curve-area under the curve (ROC-AUC) were used to evaluate the effects of each model and choose the best prediction model. To select the optimal model, we first selected the model with an ROC-AUC>0.75 to confirm that it had a good comprehensive prediction effect. On this basis, we then selected the model with the highest sensitivity as the best prediction model, thus, ensuring that as many mothers as possible with a high risk of PPD would be detected.

### Statistical Analysis

This study used the REDCap system to build a database and SPSS version 18.0 to clean the data. The training and test sets were analyzed by the “sklearn.model_selection.train_test_split” package. The RF data were analyzed by the “sklearn.ensemble.randomforestclassifiers” package. The SVM data were analyzed by the “sklearn.svm.SVC” package. Cross-validation was performed using the “sklearn.cross_validation” package. All these packages were available in the Python 3.6 software.

## Results

### Candidate Predictors

Multimedia Appendix 1 shows the 25 candidate predictors of the subjects with and without PPD. Among the 508 subjects, 173 (34.1%) were regarded as having PPD. The average age of the pregnant women was 28.64 years (SD 4.344). The average BRS score was 3.10 (SD 0.371). The average individual monthly income of the women and their spouses was between 2000 and 5000 yuan (US $393-785). Most of the subjects had a bachelor's degree. Of the 173 women with PPD, 116 (67.1%) had positive EPDS screening results in the third trimester. Multimedia Appendix 1 shows the results of the single-factor analysis (*P*<.05).

### Feature Selection

The predictive features obtained by the expert consultation and FFS-RF methods are shown in Textbox 1. This study included a total of 25 features: 17 were selected as predictive characteristics by expert consultation method and 7 were selected by FFS-RF.

**Selected features of the two methods of feature selection in descending order.**

**Expert consultation method**

- Age
- Education
- Monthly income level
- Husband’s education
- Husband’s monthly income level
- Marital satisfaction
- Sexual, psychological, or physical spousal abuse
- Childhood abuse history
- Premenstrual syndrome-mood instability
- Premenstrual syndrome-sleep changes
- Depression history of woman
- Depression history of family members
- Other mental illness history of woman
- Other mental illness history of family members
- Mother’s menopausal symptoms
- Level of psychological resilience
- Depressive symptoms in the third trimester

**Random forest-based filter feature selection**

- Level of psychological resilience
- Depressive symptoms in first trimester
- Monthly income level
- Husband’s monthly income level
- Husband’s education
- Education
- Mother’s menopausal symptoms

### Model Effects

PPD prediction models were established using the RF and SVM modeling applied to the training data set, using the feature sets constructed through our two feature selection methods. The optimal parameters of each model are shown in Table 2. After five-fold cross-validation, we found that when n_estimator=200, max_features=sqrt, and criterion=entropy, the model built using the RF algorithm and expert consultation method (E-RF) had the best sensitivity. When n_estimator=200, criterion=gini, and max_features=auto, the model built using the RF algorithm and FFS-RF method (F-RF) had the best sensitivity. Therefore, the software default setting was max_features=auto. With the SVM algorithm, regardless of the feature selection strategy, the kernel function with the highest model sensitivity was a linear kernel function.

**Figure 2:**
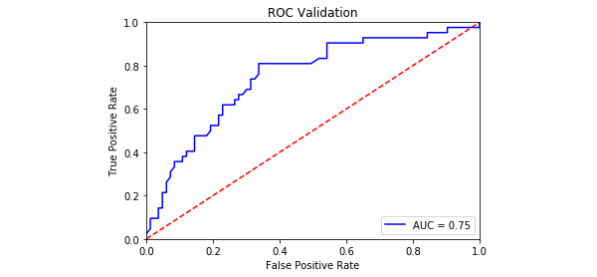
The receiver operating characteristic curve of E-RF. AUC: area under the curve; ROC: receiver operating characteristic.

**Figure 3:**
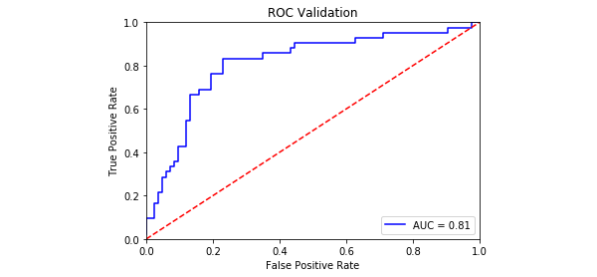
The receiver operating characteristic curve of E-SVM. AUC: area under the curve; ROC: receiver operating characteristic.

**Figure 4:**
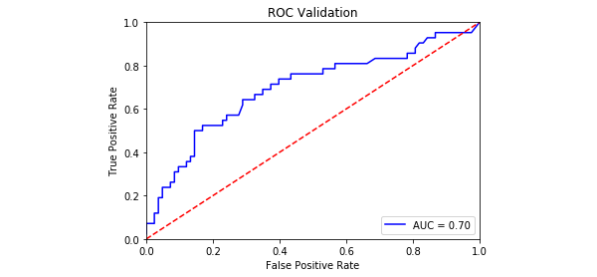
The receiver operating characteristic curve of F-RF. AUC: area under the curve; ROC: receiver operating characteristic.

**Figure 5:**
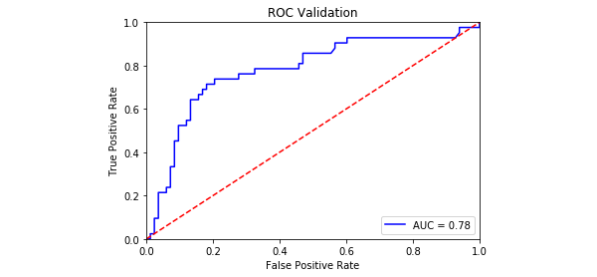
The receiver operating characteristic curve of F-SVM. AUC: area under the curve; ROC: receiver operating characteristic curve.

The model evaluation index is shown in Table 4, and the ROC curves for the four PPD models are shown in Figures 2-5. The SVM models had a slightly lower classification rate as well as a significantly higher sensitivity than the RF models. No significant differences in the specificity of each prediction model were observed. Both the positive predictive and negative predictive values of the SVM models were significantly higher than those of the RF models. With regard to feature selection, the geometric mean value for the expert consultation method was slightly higher than that of the FFS-RF. The ROC-AUC value under the SVM was slightly higher than under the RF. In summary, among the four models tested, F-SVM was the optimal model.

**Table 4:** Test data sets for each model evaluation index.

Items	E-RF^a^	E-SVM^b^	F-RF^c^	F-SVM^d^
Misclassification rate	0.28	0.20	0.27	0.22
Sensitivity	0.48	0.68	0.48	0.69
Specificity	0.86	0.87	0.86	0.83
Positive predictive value	0.63	0.72	0.63	0.68
Negative predictive value	0.76	0.84	0.76	0.84
Geometric mean	0.84	0.76	0.64	0.76
ROC-AUC^e^	0.75	0.81	0.70	0.78

^a^E-RF: model built using the random algorithm and expert consultation method.

^b^E-SVM: model built using the support vector machine algorithm and expert consultation method.

^c^F-RF: model built using the random forest algorithm and random forest-based filter feature selection method.

^d^F-SVM: model built using the support vector machine algorithm and Random forest-based filter feature selection method.

^e^ROC-AUC: receiver operating characteristic curve-area under the curve.

**Figure 6:**
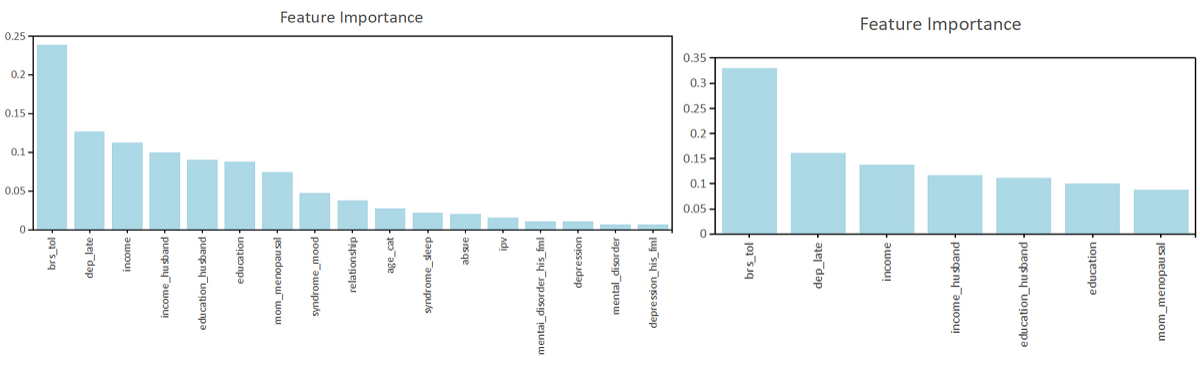
The relative feature importance rankings of the E-RF and the F-RF based on the two feature selection methods.

The features selected by the expert consultation method and FFS-RF method were put into the E-RF and F-RF models, respectively. The importance of the features was ranked as shown in Figure 6. The importance of mental elasticity in the model is significantly higher than other factors. Symptoms of depression in late pregnancy was the second most important predictor. Income levels were also important predictors of PPD. There was no significant difference in the importance of each factor to PPD. The top most important features in these two models are shown in Textbox 2.

**Top features according to the E-RF and F-RF in descending order.**

**Model built using the random forest algorithm and expert consultation method**

1. Level of psychological resilience
2. Depressive symptoms in the third trimester
3. Monthly income level
4. Husband’s education
5. Education
6. Husband’s monthly income level
7. Mother’s menopausal symptoms
8. Premenstrual syndrome-mood instability
9. Marital satisfaction
10. Age

**Model built using the random forest algorithm and random forest-based filter feature selection method**

1. Level of psychological resilience
2. Depressive symptoms in early pregnancy
3. Monthly income level
4. Husband’s monthly income level
5. Husband’s education
6. Education
7. Mother’s menopausal symptoms

## Discussion

We compared four PPD prediction models and provided a reference for the application of ML in PPD. Compared with the expert consultation method approach, the FFS-RF method identified fewer predictive factors. We found that the F-SVM model was the best model. The strongest predictive factor was the psychological resilience of pregnant women.

Between the expert consultation method and FFS-RF method, the latter selected far fewer predictive factors. Furthermore, there was no significant difference between the two methods in terms of their effects on model performance, indicating that the FFS-RS method could reduce dimensions and improve the efficiency of the algorithmic function without changing model predictive performance. The reduction in the number of predictive factors means that the burden of collecting information is reduced, making the model easier to implement and popularize, especially in busy obstetric clinics.

The SVM was chosen as the better algorithm, as it showed higher sensitivity than the RF algorithm (E-SVM=0.67, F-SVM=0.69, E-RF=0.48, F-RF=0.48). SVM had a clear advantage over RF in processing our research data, and the smaller sample size may be the main reason for this finding. Previous research on depression suggested that sample size is a key factor affecting the performance of ML models. When the sample size is small, SVM can avoid overfitting while providing efficient computing time and produces better prediction results in depression [29,30]. Our results also support this view. Therefore, we believe that when the data set is small, SVM is more practical than RF in prediction research for PPD. Several previous studies used the SVM algorithm to make PPD predictions. Jiménez [13] collected data on postpartum women from seven Spanish hospitals and used the EPDS score as the outcome indicator to train a PPD prediction model based on SVM. Sriraam [15] used social media as a data source and, based on the mental health data of 173 mothers, a SVM-based PPD prediction model was established. De Choudhury [31] developed a SVM model to identify high-risk emotions and behaviors predictive of PPD using the content of Twitter posts. As these studies either target different populations or use different methods to detect the occurrence of PPD, the model prediction effects cannot be easily compared. However, the results of the optimal F-SVM model in our study are within range (sensitivity=0.69, ROC-AUC=0.78) and consistent with the findings of previous studies (sensitivity=0.56-0.78, ROC-AUC=0.63-0.81) [13,15,31]. Due to the negative effects of PPD on mothers and infants [32,33], such as the negative effects on the physical and mental health of mothers, the closeness of the mother-infant bond, and infant development, it is important to have a model with high sensitivity while maintaining a high ROC-AUC value. The selection of indicators in evaluating depression prediction models varies across studies. For example, Sriraam [15] and De Choudhury [31] emphasized the accuracy of the model's prediction of PPD. Jiménez [13] emphasized model sensitivity and specificity. The balance between them is the geometric mean. The ROC-AUC is also widely used to evaluate the comprehensive performance of a model [14,15]. Our evaluation criteria provide a reference for prediction research for screening purposes, but the approach may be different in research studies.

We found that the top 3 most important predictors in the models were psychological resilience, depression during the third trimester, and monthly income level. First, psychological resilience is the most important factor in the prediction of PPD, which can be attributed to the protective effect of psychological elasticity. Pregnancy and childbirth are a challenging time for women emotionally and physiologically, and the mother's body and mind are under greater stress [15]. Previous research has shown that psychological resilience as an important regulatory process can enable people to recover from and adapt to stress and life events, reducing the occurrence of adverse outcomes [34-36]. Our results also support the findings of Lu [37], who found that the level of psychological elasticity was negatively correlated with the occurrence of PPD. Second, the results regarding depression in the third trimester are consistent with most previous studies. Depression in the third trimester is associated with PPD [9,38,39]. A review by Robertson [5] mentioned that “depression and anxiety during pregnancy are the strongest predictors of PPD”. Mora's [40] research suggests that depression in the third trimester may continue to develop into the postpartum period. Third, the monthly income levels remain important factors affecting PPD, which supports Rhonda's [41] findings that mothers with low income levels faced obstacles in using mental health resources and were more likely to be frustrated. Epidemiological studies of PPD worldwide have also found that the incidence in developing countries is higher than that in developed countries [42].

The identification of these predictors also reveals the different aspects of PPD risk factors. A pregnant woman's psychological elasticity may reflect her personality traits. Depression in the third trimester may be a special symptom accompanying pregnancy. The income of a pregnant woman and her partner reflects the stability and coping resources available to them. It indicates that PPD risk should be assessed based on a combination of individual long-term, short-term, and environmental characteristics.

This study has several limitations. First, there was potential selection bias. Women who were not lost to follow-up might have had a greater awareness of mental health services. Second, the 50% loss to follow-up and small sample size may have negatively affected the applicability of the PPD model, indicating that more extensive validation is required. Third, a larger number of potential predictive factors would have been useful. Further studies should develop different PPD models using other ML algorithms and data from different sources as well as incorporating additional cultural factors to expand the application of the PPD models.

## Appendix

Multimedia Appendix 1 Comparison of candidate predictors in the sample of pregnant women (N=508).

Multimedia Appendix 2 Comparison of demographic characteristics, including data sets of 618 pregnant women lost in the cohort and 508 mothers who left the cohort study after childbirth.

Multimedia Appendix 3 Definitions and coding of analyzed variables.

## Abbreviations

BRS: Brief Resilience Scale
E-RF: model built using the random forest algorithm and expert consultation method
E-SVM: model built using the support vector machine algorithm and expert consultation method
EPDS: Edinburgh Postnatal Depression Scale
F-RF: model built using the random forest algorithm and Random forest-based filter feature selection method
F-SVM: model built using the support vector machine algorithm and Random forest-based filter feature selection method
FFS-RF: random forest-based filter feature selection
GAD-7: Generalized Anxiety Disorder-7
ML: machine learning
PPD: postpartum depression
PSQI: Pittsburgh Sleep Quality Index
RF: random forest
ROC-AUC: receiver operator curve-area under the curve
SSRS: Social Support Rating Scale
SVM: support vector machine.
