# Supplementary material for: Machine Learning Models for the Prediction of Postpartum Depression: Application and Comparison Based on a Cohort Study
Source: JMIR Med Inform. 2020 Apr 30;8(4):e15516. doi: 10.2196/15516 (PMC7226048; doi:10.2196/15516)
Supplement: Multimedia Appendix 1 [file medinform_v8i4e15516_app1.docx]

Comparison of candidate predictors in the sample of pregnant women (N=508).

| Items | | Women without PPD^a^ (n=335) | Women with PPD (n=173) |  | *P* value |
| --- | --- | --- | --- | --- | --- |
| Age (years), mean (SD) | | 28.7 (4.5) | 28.5 (4.0) | 0.7 | .49 |
| **Education, n (%)** | | | | -0.6 | .54 |
|  | Junior high school or below | 29 (8.7) | 19 (11.0） |  |  |
|  | High school | 77 (23.1) | 40 (23.1) |  |  |
|  | Bachelor’s | 198 (59.3) | 99 (57.2) |  |  |
|  | Master’s degree or above | 30 (9.0) | 15 (8.7) |  |  |
| **Education of husband, n (%)** | | | | -0.2 | .87 |
|  | Junior high school or below | 42 (12.6) | 20 (11.9) |  |  |
|  | High school | 79 (23.7) | 36 (21.4) |  |  |
|  | Bachelor’s | 177 (53.2) | 99 (58.9) |  |  |
|  | Master’s degree and above | 35 (10.5) | 13 (7.7) |  |  |
| **Monthly income level (yuan), n (%)** | | | | -1.7 | .09 |
|  | 0 | 80 (24.3) | 56 (33.3) |  |  |
|  | >0 and <2000 | 20 (6.1) | 9 (5.4) |  |  |
|  | ≥2000 and <5000 | 178 (54.1) | 78 (46.4) |  |  |
|  | ≥5000 and <10000 | 41 (12.5) | 22 (13.1) |  |  |
|  | ≥10000 | 10 (3.0) | 3 (1.8) |  |  |
| **Monthly income level of husband (yuan), n (%)** | | | | -0.6 | .52 |
|  | 0 | 5 (1.5) | 6 (3.5) |  |  |
|  | >0 and <2000 | 12 (3.6) | 2 (1.2) |  |  |
|  | ≥2000 and <5000 | 158 (47.4) | 75 (44.1) |  |  |
|  | ≥5000 and <10000 | 119 (35.7) | 67 (39.4) |  |  |
|  | ≥10000 | 39 (11.7) | 20 (11.8) |  |  |
| **Occupation, n (%)** | | | | 10.8 | .03 |
|  | Public officials | 80 (24.2) | 28 (16.4) |  |  |
|  | Corporation management | 59 (17.8) | 33 (19.3) |  |  |
|  | In business (self-employed) | 52 (15.7) | 20 (11.7) |  |  |
|  | Unemployed | 73 (22.1) | 58 (33.9) |  |  |
|  | Others | 67 (20.2) | 32 (18.7) |  |  |
| **Marital satisfaction, n (%)** | | | | -3.3 | <.001 |
|  | Satisfied | 294 (88.6) | 133 (77.3) |  |  |
|  | Basically satisfied | 38 (11.4) | 38 (22.1) |  |  |
|  | Dissatisfied | 0 (0.0) | 1 (0.6) |  |  |
| **First pregnancy, n (%)** | | | | 0.1 | .70 |
|  | No | 231 (70.4) | 119 (68.8) |  |  |
|  | Yes | 97 (29.6) | 54 (31.2) |  |  |
| **Folic acid intake before this pregnancy, n (%)** | | | | 0.0 | .90 |
|  | No | 127 (38.0) | 64 (37.4) |  |  |
|  | Yes | 207 (62.0) | 107 (62.6) |  |  |
| **Premenstrual syndrome-mood instability, n (%)** | | | | 20.0 | <.001 |
|  | No | 256 (76.4) | 99 (57.2) |  |  |
|  | Yes | 79 (23.6) | 74 (42.8) |  |  |
| **Premenstrual syndrome-sleep changes, n (%)** | | | | 0.7 | .42 |
|  | No | 316 (94.3) | 160 (92.5) |  |  |
|  | Yes | 19 (5.7) | 13 (7.5) |  |  |
| **Depression history, n (%)** | | | | 3.3 | .07 |
|  | No | 224 (96.1) | 107 (91.5) |  |  |
|  | Yes | 9 (3.9) | 10 (8.5) |  |  |
| **Other mental illness history, n (%)** | | | | 0.1 | .70 |
|  | No | 323 (99.1) | 167 (98.2) |  |  |
|  | Yes | 3 (0.9) | 3 (1.8) |  |  |
| **Depression history in family members, n (%)** | | | | 0.9 | .64 |
|  | No | 317 (94.9) | 162 (94.2) |  |  |
|  | Yes | 8 (2.4) | 3 (1.7) |  |  |
|  | Unsure | 9 (2.7) | 7 (4.1) |  |  |
| **Other mental illness history in family members, n (%)** | | | | N/A^b^ | .18 |
|  | No | 325 (97.3) | 164 (95.3) |  |  |
|  | Yes | 4 (1.2) | 1 (0.6) |  |  |
|  | Unsure | 5 (1.5) | 7 (4.1) |  |  |
| **Mother's menopausal symptoms, n (%)** | | | | 10.0 | .007 |
|  | No | 198 (59.3) | 76 (44.4) |  |  |
|  | Yes | 58 (17.4) | 41 (24.0) |  |  |
|  | Others | 78 (23.1) | 54 (31.6) |  |  |
| **Suffered sexual, psychological, or physical violence at an early age, n (%)** | | | | 10.7 | <.001 |
|  | No | 324 (96.7) | 155 (89.6) |  |  |
|  | Yes | 11 (3.3) | 18 (10.4) |  |  |
| **Suffering from sexual, psychological, or physical violence from husband, n (%)** | | | | 9.3 | .002 |
|  | No | 310 (92.5) | 145 (83.8) |  |  |
|  | Yes | 25 (7.5) | 28 (16.2) |  |  |
| **Results of EPDS^c^ (first trimester), n (%)** | | | | 67.9 | <.001 |
|  | Negative | 268 (80.0) | 76 (43.9) |  |  |
|  | Positive | 67 (20.0) | 97 (56.1) |  |  |
| **Results of EPDS (second trimester), n (%)** | | | | 65.5 | <.001 |
|  | Negative | 254 (75.8) | 68 (39.3) |  |  |
|  | Positive | 81 (24.2) | 105 (60.7) |  |  |
| **Results of EPDS (third trimester), n (%)** | | | | 95.5 | <.001 |
|  | Negative | 259 (77.3) | 57 (32.9) |  |  |
|  | Positive | 76 (22.7) | 116 (67.1) |  |  |
| Scores of BRS^d^, mean (SD) | | 3.1 (0.3) | 3.1 (0.4) | -1.2 | .25 |
| **Scores of PSQI^e^, mean (SD)** | | | | | |
|  | Subjective sleep quality | 1.0 (0.6) | 1.2 (0.6) | -5.3 | <.001 |
|  | Sleep latency | 1.0 (0.8) | 1.4 (0.9) | -5.2 | <.001 |
|  | Sleep duration | 0.2 (0.6) | 0.39 (0.7) | -2.5 | .01 |
|  | Habitual sleep efficiency | 0.4 (0.8) | 0.7 (0.9) | -3.6 | <.001 |
|  | Sleep disturbance | 1.2 (0.5) | 1.4 (0.6) | -4.9 | <.001 |
|  | Sleep medication | 0.0 (0.0) | 0.00 (0.0) | N/A | N/A |
|  | Daytime dysfunction | 1.3 (0.9) | 1.5 (0.9) | -2.7 | .006 |
|  | Global PSQI score | 5.0 (2.5) | 6.6 (2.9) | -5.9 | <.001 |
| **Scores of SSRS^f^, mean (SD)** | | | | | |
|  | Objective support | 10.3 (2.5) | 9.2 (2.4) | 4.3 | <.001 |
|  | Subjective support | 21.8 (3.7) | 19.8 (4.3) | 5.1 | <.001 |
|  | Use of support | 8.2 (1.9) | 7.9 (2.0) | 1.8 | .08 |
|  | Total scores | 40.3 (6.1) | 36.9 (7.0) | 5.5 | <.001 |
| **Results of GAD-7^g^, n (%)** | | | | 2.7 | .10 |
|  | Negative | 324 (97.9) | 160 (95.2) |  |  |
|  | Positive | 7 (2.1) | 8 (4.8) |  |  |

^a^PPD: postpartum depression.

^b^N/A: not applicable^.^

^c^EPDS: Edinburgh Postnatal Depression Scale.

^d^BRS: Brief Resilience Scale.

^e^PSQI: Pittsburgh Sleep Quality Index.

^f^SSRS: Social Support Rating Scale.

^g^GAD-7: Generalized Anxiety Disorder-7 Item.
