# Supplementary material for: Machine Learning Models for the Prediction of Postpartum Depression: Application and Comparison Based on a Cohort Study
Source: JMIR Med Inform. 2020 Apr 30;8(4):e15516. doi: 10.2196/15516 (PMC7226048; doi:10.2196/15516)
Supplement: Multimedia Appendix 2 [file medinform_v8i4e15516_app2.docx]

| **Appendix 2.** Comparison of demographic characteristics, including data sets of 618 pregnant women lost in the cohort and 508 mothers after childbirth who still left in the cohort study. | | | | | | | |  |
| --- | --- | --- | --- | --- | --- | --- | --- | --- |
| Item | | Lost pregnant women(n=618) | | Postpartum(n=508) | |  | *P* value |  |
|  |  | n (%) | | n(%) | |  |  |  |
| **Age(years), (Mean, SD)** | | 28.94(4.679) | | 28.64(4.344) | | 1.1 | .29 |  |
| **Education degree** | |  | |  | |  |  |  |
|  | Junior high school and below | 79(13.0) | | 48(9.5） | | -1.5 | .14 |  |
|  | High school | 141(23.2) | | 117(23.1) | |  |  |  |
|  | Bachelor’s | 340(55.8) | | 297(58.6) | |  |  |  |
|  | Master’s degree and above | 49(8.0) | | 45(8.9) | |  |  |  |
| **Education degree(husband)** | |  | |  | |  |  |  |
|  | Junior high school and below | 82(13.5) | | 62(12.4) | | -1.0 | .34 |  |
|  | High school | 147(24.2) | | 115(23.0) | |  |  |  |
|  | Bachelor’s | 327 (53.9) | | 276(55.1) | |  |  |  |
|  | Master’s degree and above | 51(8.4) | | 48(9.6) | |  |  |  |
| **Income level** | |  | |  | |  |  |  |
|  | ¥0 | 169(28.3) | | 136(27.4) | | -1.3 | .20 |  |
|  | >¥0 and <¥2000 | 36(6.0) | | 29(5.8) | |  |  |  |
|  | ≥¥2000 and <¥5000 | 255(42.6) | | 256(51.5) | |  |  |  |
|  | ≥¥5000 and <¥10000 | 119(19.9) | | 63(12.7) | |  |  |  |
|  | ≥¥10000 | 19(3.2) | | 13(2.6) | |  |  |  |
| **Appendix 1.**Comparison of demographic characteristics, including data sets of 618 pregnant women lost in the cohort and 508 mothers after childbirth who still left in the cohort study. | | | | | | | |  |
| Item | | Lost pregnant women(n=618) | | Postpartum(n=508) | |  | *P* value |  |
|  |  | n (%) | | n(%) | |  |  |  |
| **Income level(husband)** | |  | |  | |  |  |  |
|  | ¥0 | 14(2.3) | | 11(2.2) | | -2.0 | .50 |  |
|  | >¥0 and <¥2000 | 18(2.9) | | 14(2.8) | |  |  |  |
|  | ≥¥2000 and <¥5000 | 256(41.1) | | 238(46.9) | |  |  |  |
|  | ≥¥5000 and <¥10000 | 231(37.4) | | 186(36.6) | |  |  |  |
|  | ≥¥10000 | 99(16.0) | | 59(11.6) | |  |  |  |
| **occupation** | |  | |  | |  |  |  |
|  | Public officials | 128(21.3) | | 108(21.5) | | -0.4 | .68 |  |
|  | Corporation management | 102(16.9) | | 92(18.3) | |  |  |  |
|  | In business (self-employed) | 95(15.8) | | 72(14.3) | |  |  |  |
|  | Unemployed | 165(24.8) | | 137(26.1) | |  |  |  |
|  | Others | 128(21.3) | | 99(19.7) | |  |  |  |
| **Marital satisfaction** | |  | |  | |  |  |  |
|  | Satisfied | 478(78.7) | | 427(84.7) | | -1.0 | .34 |  |
|  | Basically satisfied | 125(20.6) | | 76(15.1) | |  |  |  |
|  | Dissatisfied | 4(0.7) | | 1(0.2) | |  |  |  |
| **First pregnancy** | |  | |  | |  |  |  |
|  | No | | 453(76.1) | | 350(69.9) | 5.5 | .02 | |
|  | Yes | | 142(23.9) | | 151(30.1) |  |  |  |
| **Appendix 1.**Comparison of demographic characteristics, including data sets of 618 pregnant women lost in the cohort and 508 mothers after childbirth who still left in the cohort study. | | | | | | | |  |
| Item | | Lost pregnant women(n=618) | | Postpartum(n=508) | |  | *P* value |  |
|  |  | n (%) | | n(%) | |  |  |  |
| **Folic acid intake before this pregnancy** | |  | |  | |  |  |  |
|  | No | | 222(36.8) | | 191(37.8) | 0.1 | .71 |  |
|  | Yes | | 382(63.2) | | 314(62.2) |  |  |  |
| **Premenstrual syndrome -mood instability** | |  | |  | |  |  |  |
|  | No | 429(69.4) | | 355(69.9) | | 0.02 | .87 |  |
|  | Yes | 189(30.6) | | 153(30.1) | |  |  |  |
| **Premenstrual syndrome-sleep changes** | |  | |  | |  |  |  |
|  | No | 572(92.6) | | 476(93.7) | | 0.6 | .45 |  |
|  | Yes | 46(7.4) | | 32(6.3) | |  |  |  |
| **Depression history** | |  | |  | |  |  |  |
|  | No | 602(97.4) | | 489(96.3) | | 1.2 | .27 |  |
|  | Yes | 16(2.6) | | 19(3.7) | |  |  |  |
| **Other mental illness history** | |  | |  | |  |  |  |
|  | No | 581(99.1) | | 490(98.8) | | 0.4 | .56 |  |
|  | Yes | 5(0.8) | | 6(1.2) | |  |  |  |
| **Appendix 1.**Comparison of demographic characteristics, including data sets of 618 pregnant women lost in the cohort and 508 mothers after childbirth who still left in the cohort study. | | | | | | | |  |
| Item | | Lost pregnant women(n=618) | | Postpartum(n=508) | |  | *P* value |  |
|  |  | n (%) | | n(%) | |  |  |  |
| **Depression history of other family members** | |  | |  | |  |  |  |
|  | No | 572(94.1) | | 479(94.7) | | 3.6 | .17 |  |
|  | Yes | 7(1.2) | | 11(2.2) | |  |  |  |
|  | Not clear | 29(4.8) | | 16(3.2) | |  |  |  |
| **Other mental illness history of other family members** | |  | |  | |  |  |  |
|  | No | 583(96.0) | | 489(96.6) | | 0.7 | .72 |  |
|  | Yes | 5(0.8) | | 5(1.0) | |  |  |  |
|  | Not clear | 19(3.1) | | 12(2.4) | |  |  |  |
| **Mother's menopausal symptoms** | |  | |  | |  |  |  |
|  | No | 340(56.1) | | 274(54.3) | | -0.4 | .71 |  |
|  | Yes | 105(17.3) | | 99(19.6) | |  |  |  |
|  | Others | 161(26.6） | | 132(26.1) | |  |  |  |
| **Suffered sexual/psychological/physical violence in early age** | |  | |  | |  |  |  |
|  | No | 604(97.7) | | 479 (94.3) | | 9.0 | .003 |  |
