# Supplementary material for: Machine Learning Models for the Prediction of Postpartum Depression: Application and Comparison Based on a Cohort Study
Source: JMIR Med Inform. 2020 Apr 30;8(4):e15516. doi: 10.2196/15516 (PMC7226048; doi:10.2196/15516)
Supplement: Multimedia Appendix 3 [file medinform_v8i4e15516_app3.docx]

**Appendix 3.** Definitions and coding of Analyzed Variables

| **Variables** | **Definitions** |
| --- | --- |
| **Education degree** | Junior high school and below=1 |
|  | High school=2 |
|  | Bachelor’s=3 |
|  | Master’s degree and above=4 |
| **Income level** | ¥0 = 1 |
|  | ＞¥0 and ＜¥2000 = 2 |
|  | ≥¥2000 and ＜¥5000 = 3 |
|  | ≥¥5000 and ＜¥10000 = 4 |
|  | ≥¥10000 = 5 |
| **Occupation** | Public officials = 1 |
|  | Corporation managers = 2 |
|  | In business (self-employed) = = 3 |
|  | Unemployed =4 |
|  | Others = 5 |
| **Marital satisfaction** | Satisfied = 1 |
|  | Basically satisfied =2 |
|  | Dissatisfied =3 |
| **First pregnancy** | No=1, Yes= 2 |
| **Folic acid intake before this pregnancy** | No=1, Yes= 2 |
| **Appendix 2.** Definitions and coding of Analyzed Variables | |
| **Variables** | **Definitions** |
| **Premenstrual syndrome-mood instability** | No=1, Yes=2 |
| **Premenstrual syndrome-sleep changes** | No=1, Yes=2 |
| **Premenstrual syndrome** | Headache, backache, breast swelling, constipation, limb edema, etc. (physical symptoms) = 1 |
|  | Irritability, anxiety, depression and emotional instability = 2 |
|  | Fatigue = 3 |
|  | Changes in sleep and sexual desire = 4 |
|  | Lack of concentration = 5 |
|  | Neurotic = 6 |
|  | No symptoms above, but other discomfort = 7 |
|  | No discomfort = 8 |
|  | When participants choose 2, Premenstrual syndrome -mood instability =1 (Yes), otherwise =0 (No) |
|  | When participants choose 4, Premenstrual syndrome -sleep changes =1 (Yes), otherwise =0 (No) |
| **Depression history** | No=1, Yes=2 |
| **Other mental illness history** | No=1, Yes=2 |
| **Depression history of other family members** | No=1, Yes=2, Not clear=3 |
| **Other mental illness history of other family members** | No=1, Yes=2, Not clear=3 |
| **Appendix 2.** Definitions and coding of Analyzed Variables | |
| **Variables** | **Definitions** |
| **Mother's menopausal symptoms** | No=1, Yes=2, Others=3 |
| **Suffered sexual/ psychological /physical violence in early age** | No=1, Yes=2 (It’s defined as be abused before you 16, including but not limited to severe beatings, mental torture, abuse, hatred, long-term neglect, sexual assault, etc., as long as one of the above exists, or although the above acts did not occur but you feel abused, tick "yes") |
| **Suffering from sexual/psychological/physical violence from husband** | No=1, Yes=2 (The violence is including sexual/psychological/physical violence, which is specific in that 1)partner pushed, hit or kicked pregnant without joking;2)used force / threatened to insist on having sex with pregnant when she didn't want to;3) partner ignored pregnant for a long time/ completely;4) partner insulted pregnant. As long as one of the above exists, tick "yes") |
| **Results of EPDS^a^ (first trimester)** | Negative (The score of EPDS in T1<10) =0, Positive (The score of EPDS in T1≥10) =1 |
| **Results of EPDS (second trimester)** | Negative (The score of EPDS in T2<10) =0, Positive (The score of EPDS in T2≥10) =1 |
| **Results of EPDS (third trimester)** | Negative (The score of EPDS in T3<10) =0, Positive (The score of EPDS in T3≥10) =1 |
| **Results of GAD-7^b^** | Negative=0 (Sores of GAD-7 is lower than 10), Positive=1(Scores of GAD-7 is higher than 10, including 10) |
